# A High-Density Genetic Map of Tetraploid *Salix matsudana* Using Specific Length Amplified Fragment Sequencing (SLAF-seq)

**DOI:** 10.1371/journal.pone.0157777

**Published:** 2016-06-21

**Authors:** Jian Zhang, Huwei Yuan, Min Li, Yujuan Li, Ying Wang, Xiangjian Ma, Yuan Zhang, Feng Tan, Rongling Wu

**Affiliations:** 1 Center for Computational Biology, College of Biological Sciences and Technology, Beijing Forestry University, Beijing, China; 2 National Engineering Laboratory for Forest Tree Breeding, Key Laboratory for Genetics and Breeding of Forest Trees and Ornamental Plants of Ministry of Education, College of Biological Sciences and Technology, Beijing Forestry University, Beijing, China; 3 Jiangsu Riverine Institute of Agricultural Sciences, Rugao, Jiangsu, China; 4 Center for Statistical Genetics, Pennsylvania State University, Hershey, United States of America; The State University of New York at Stony Brook, UNITED STATES

## Abstract

As a salt-tolerant arbor tree species, *Salix matsudana* plays an important role in afforestation and greening in the coastal areas of China. To select superior *Salix* varieties that adapt to wide saline areas, it is of paramount importance to understand and identify the mechanisms of salt-tolerance at the level of the whole genome. Here, we describe a high-density genetic linkage map of *S*. *matsudana* that represents a good coverage of the Salix genome. An intraspecific F_1_ hybrid population was established by crossing the salt-sensitive “Yanjiang” variety as the female parent with the salt-tolerant “9901” variety as the male parent. This population, along with its parents, was genotyped by specific length amplified fragment sequencing (SLAF-seq), leading to 277,333 high-quality SLAF markers. By marker analysis, we found that both the parents and offspring were tetraploid. The mean sequencing depth was 53.20-fold for “Yanjiang”, 47.41-fold for “9901”, and 11.02-fold for the offspring. Of the SLAF markers detected, 42,321 are polymorphic with sufficient quality for map construction. The final genetic map was constructed using 6,737 SLAF markers, covering 38 linkage groups (LGs). The genetic map spanned 5,497.45 cM in length, with an average distance of 0.82 cM. As a first high-density genetic map of *S*. *matsudana* constructed from salt tolerance-varying varieties, this study will provide a foundation for mapping quantitative trait loci that modulate salt tolerance and resistance in Salix and provide important references for molecular breeding of this important forest tree.

## Introduction

Willows, the general name of species in *Salix* (*Salicaceae*), are deciduous trees or shrubs distributed mainly in the temperate and frigid zones of the Northern Hemisphere. There are > 500 willow species worldwide, about half of which can be found in China. Willows are important tree species for energy production, afforestation, and greening [1–3]. Some willow varieties, including *S*. *matsudana* and *Salix psammophila*, have been receiving increasing attention because of their salt tolerance [4–8]. Some varieties of *S*. *matsudana* have become potential strategic resources in coastal forestry exploitations of China [9, 10]. To date, the responses of *S*. *matsudana* to salt stress have been studied at the levels of physiology [10], gene expression [9], and miRNA expression [11]. However, the mechanisms of salt tolerance at the whole genome level of *S*. *matsudana* has not been extensively explored thus far.

Genetic maps that are constructed according to the linkage relationships among genetic markers at the whole genome level are the basis for quantitative trait locus (QTL) mapping, map-based gene cloning, comparative genomics, and marker-assisted breeding. Willows have relatively high recombination rates and low levels of linkage disequilibrium (LD) [12], which makes them suitable for genetic mapping. The first genetic linkage map of Salix was constructed using a population of 87 hybrids derived from a cross between "Björn" (the male hybrid clone of *Salix viminalis* × *Salix schwerinii*) and "78183" (the female clone of *Salix viminalis*). The map consisted of 325 amplified fragment length polymorphisms (AFLP) and 38 restriction fragment length polymorphisms (RFLP) markers with an average density of markers of 14 cM [13]. Later, two linkage maps of *Salix* containing 495 single nucleotide polymorphisms (SNP) and 221 AFLP markers were consrtucted, with the average distances of 5.0 and 8.1 cM, respectively [14]. All these maps were constructed using diploid (2n = 38) willows and were of low density, with the average marker densities of 5.0~14 cM. Barcaccia et al. [15] have successfully constructed a genetic map for tetraploid (2n = 4x = 76) *Salix*, but its marker density needs to be increased for a better understanding of the genome structure and organization of tetraploid *Salix*.

Single nucleotide polymorphisms (SNP) represent DNA sequence variation among individuals caused by single base mutations. SNP markers have become a powerful tool in genetics due to their abundant and even distribution. Recent advances in next-generation sequencing (NGS) technologies have provided enormous impetus for the rapid development and extensive application of SNP markers [16]. Technologies that can develop SNP markers in a short time include complexity reduction of polymorphic sequences (CroPS) [17], restriction-site-associated DNA sequencing (RAD-seq) [18], and genotyping-by-sequencing [19]. Specific length amplified fragment sequencing (SLAF-seq) is a newly developed NGS technology that can be used to rapidly develop SNP markers by constructing a SLAF-seq library [20]. Development of SNP markers and construction of high-density genetic maps based on SLAF-seq have been applied to a number of species [20–24].

In this study, “Yanjiang” (a salt-sensitive variety of *S*. *matsudana* native to the Jiangsu riverine areas of China) and “9901” (a salt-tolerant variety of *S*. *matsudana* in Shandong coastal areas of China) were used as female and male parents, respectively, and the intraspecific F_1_ hybrid population containing 3,520 individuals were obtained through controlled pollination. A total of 200 individuals, along with its parents, were selected randomly from the F_1_ population and used as the mapping population. SLAF-seq technology [20] was used to develop SLAF markers (SLAFs) and construct a high-density intraspecific genetic map of tetraploid *S*. *matsudana* constructed from salt tolerance-varying varieties. Results from this study will provide a foundation for genetic map-based QTL fine mapping, gene cloning, comparative genomics, and marker-assisted breeding of salt-tolerant related traits in this important tree species.

## Materials and Methods

### Plant Material, DNA Extraction, and Identification of Ploidy Levels

Main branches of “Yanjiang” (female parent) and “9901” (male parent) with ≥ 5 cm diameters were collected on December 15, 2013, and then cultured hydroponically in an intelligent greenhouse at 20–25°C (This work was conducted in Jiangsu Riverine Institute of Agricultural Sciences. We are members in Jiangsu Riverine Institute of Agricultural Sciences and this Institute granted us full permission of the work. This study did not involve endangered or protected species.). Mature pollens were collected from “9901” and then crossed with receptive female flowers on “Yanjiang”. A total of 3,520 F_1_ offspring were obtained and sown. Next, 200 individuals were selected randomly from the F_1_ population on Jul. 3, 2014, and used as a mapping population. Genomic DNA of the mapping population was extracted using the cationic detergent cety-ltrimethylammonium bromide (CTAB) method [25]. The extracted DNA was detected by agarose gel electrophoresis (1%) and then analyzed on the ND-1000 spectrophotometer platform (NanoDrop, Wilmington, DE, USA) for concentration and purity. Ploidy levels of the parents and offspring were examined based on flow cytometry [26] according to the method of Serapiglia et al. [27].

### Construction and Sequencing of the SLAF Library and Development of Polymorphic SLAF Markers

The genome of *Populous trichocarpa* (http://www.ncbi.nlm.nih.gov/assembly/GCF_000002775.3) was chosen as the reference genome for pre-restriction enzyme digestion according to the genome size and GC content information. *Hae*III and *Hpy*166II restriction enzymes were finally chosen to digest genomic DNA of the mapping population. After digestion by *Hae*III and *Hpy*166II, the obtained SLAFs (314–364 bp in length) of the mapping population had an A-tail added to the 3′ ends, was ligated with Dual-index sequencing adaptors, amplified by PCR, screened, and then used to construct the SLAF library of *S*. *matsudana* (for detailed processes of SLAF library construction refer to the methods of Sun et al. [20]).

SLAFs in the quality-tested library were sequenced using the Illumina HiSeq 2500 platform (Illumina, San Diego, CA, USA). The genome of *Oryza sativa*, used as a control, underwent the same treatments of library construction and sequencing as the *S*. *matsudana* mapping population to check the reliability of testing processes.

Reads of the samples were obtained by identifying Dual-index sequences. The adaptor-filtered reads were evaluated for quality and data size, and then clustered to develop SLAFs in the parents and offspring. Polymorphic SLAFs were selected according to the single nucleotide mutations (SNP markers) and insertion and deletion mutations (InDel markers).

### Construction of High-Density Genetic Maps

The high-quality polymorphic SLAFs were allocated into different linkage groups (LGs) according to their method limit of detection (MLOD) values. Genetic maps were constructed and corrected using HighMap software according to the methods of [22]. The genetic map was evaluated according to the haplotype maps, percentage of missing SLAFs, and heat maps of each LG.

## Results

### Ploidy Levels of the Parents and Offspring

**Fig 1:**
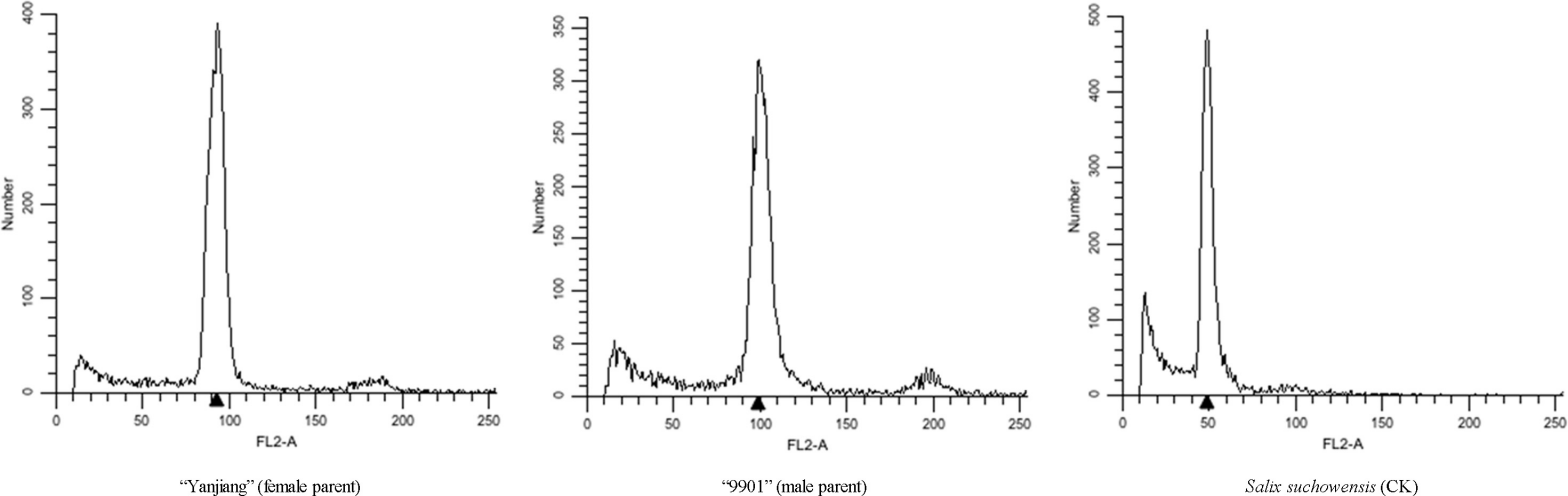
Ploidy levels of *S*. *matsudana* parents.

**Fig 2:**
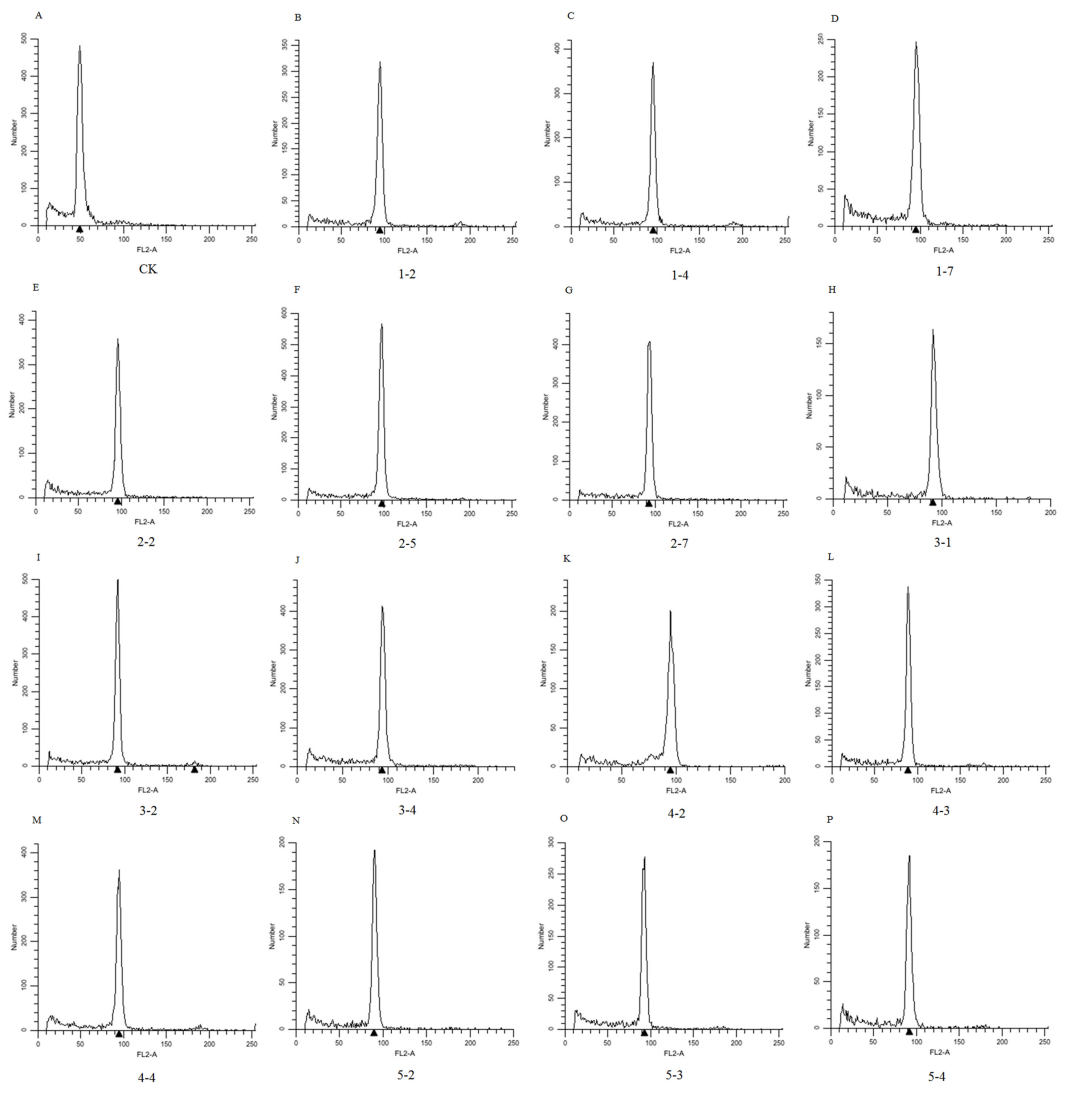
Ploidy levels of *S*. *matsudana* offspring.

*Salix suchowensis*, the already known diploid (2n = 2x = 38), was used as a control to identify ploidy levels of the parents and offspring. Our results showed that the peak values of the parents were twice that of *S*. *suchowensis*, indicating that both the female parent and male parent were tetraploid (2n = 4x = 76) (Fig 1). Similarly, the randomly selected 15 offspring in the mapping population were all tetraploid (2n = 4x = 76) (Fig 2).

### Quality of SLAF-seq Data

Evaluations of the *Oryza sativa* SLAF library showed that cleavage efficiency of the *Hae*III and *Hpy*166II restriction enzymes was 92.06%, and the paired-end reads accounted for 96.67% of all reads obtained. These results indirectly reflected that the SLAF library of *S*. *matsudana* was of high quality. DNA sequencing generated a total of 472.53 M reads. Mean Q30 percentage (the percentage of bases with sequencing values ≥ 30 in the total bases) and mean GC percentage (the percentage of G and C bases in the total bases) of the *S*. *matsudana* parents and offspring were 90.15% and ≈38%, respectively. Basic statistics of the SLAF-seq data are listed in Table 1.

**Table 1:** Basic statistics of the SLAF-seq data in *S*. *matsudana*.

Sample ID	Total reads	Q30 Percentage (%)	GC Percentage (%)
**9901 (male parent)**	14,160,572	93.98	37.59
**Yanjiang (female parent)**	13,640,990	93.84	38.03
**Average of 195 offspring**	2,223,636	90.11	38.14
**Rice (Control)**	397,584	93.26	42.23

Note: Total reads represents the number of total reads; Q30 percentage represents the percentage of bases with sequencing values ≥ 30 in the total bases; GC percentage represents the percentage of G and C bases in the total bases.

### Development of SLAF Markers

After analyzing the SLAF-seq data, five offspring with the percentage of abnormal SLAFs in total SLAFs of ≥ 0.3% were removed from the mapping population and the remaining 195 offspring and the parents were retained. In total, 277,333 SLAFs were detected, of which the average depth of the male parent, female parent, and offspring were 53.20-fold, 47.41-fold, and 11.02-fold, respectively (Table 2).

**Table 2:** Summary of sequencing depth of SLAF markers.

Sample ID	The number of SLAFs	Total depth	Average depth
**9901 (male parent)**	207,443	11,036,037	53.20
**Yanjiang (female parent)**	206,718	9,801,466	47.41
**Average of 195 offspring**	150,292	1,666,374	11.02

The 277,333 SLAFs were classified into the three categories of polymorphic, non- polymorphic, and repetitive according to differences in allele number and sequences. Of all the SLAFs obtained, 99,526 (accounting for 35.89% of the total SLAFs) were polymorphic (Table 3).

**Table 3:** Classification of SLAF markers.

Type	Polymorphic SLAFs	Non-Polymorphic SLAFs	Repetitive SLAFs	Total SLAFs
**Number**	99, 526	175,800	2,007	277, 333
**Percentage**	35.89%	63.39%	0.72%	100%

**Fig 3:**
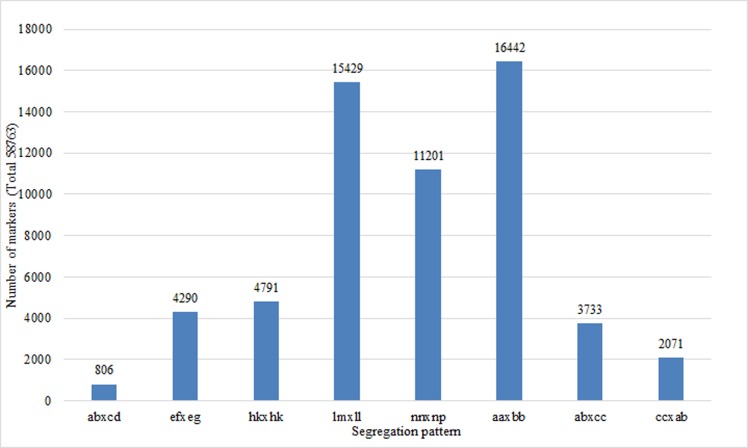
Classification of the filtered polymorphic specific length amplified fragment sequencing (SLAF) markers.

After filtering out the 99,526 polymorphic SLAFs lacking parent information, 58,763 were retained and further classified into eight segregation patterns (Fig 3). The number of SLAFs ranged from 806 to 16,442 in different patterns. The detailed distribution of the markers is shown in Fig 3.

Since F_1_ offspring were used as the mapping population, SLAFs with an aaxbb pattern were filtered. The remaining seven segregation patterns of SLAFs (42,321) were candidate markers to construct the genetic map.

### Construction of the High-Density Genetic Map

**Fig 4:**
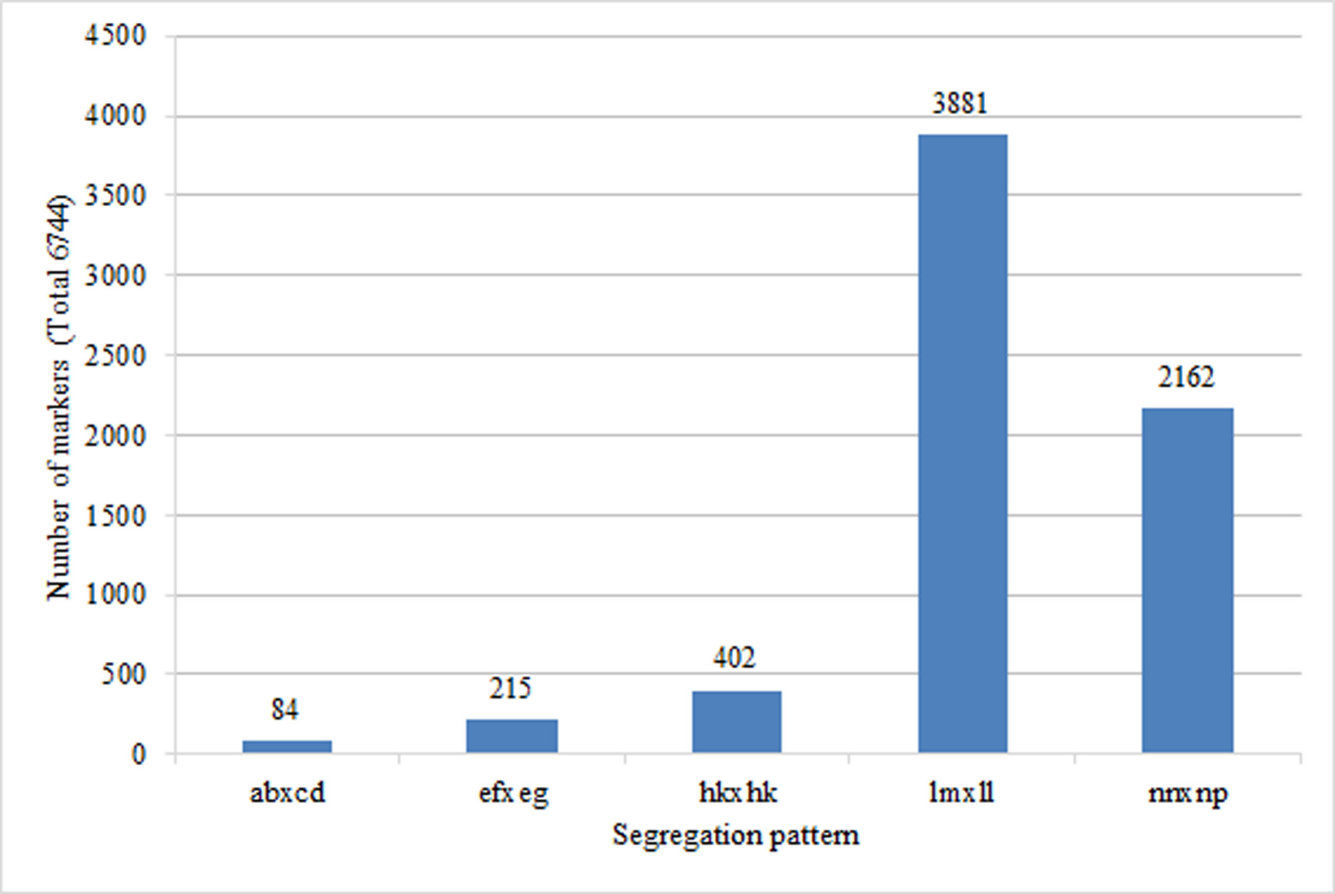
Classification of SLAF markers suitable to construct the genetic map.

To confirm the quality of the genetic map, the 42,321 polymorphic SLAFs with the following characteristics were further filtered: 1) number of SNPs ≥ 3; 2) sequencing depths of the parents ≤ 10-fold; 3) integrity ≤ 85%; 4) distorted segregation; 5) homozygous parents. Finally, 6,744 high quality SLAFs belonged to five segregation patterns (Fig 4) were suitable to construct the genetic map.

**Fig 5:**
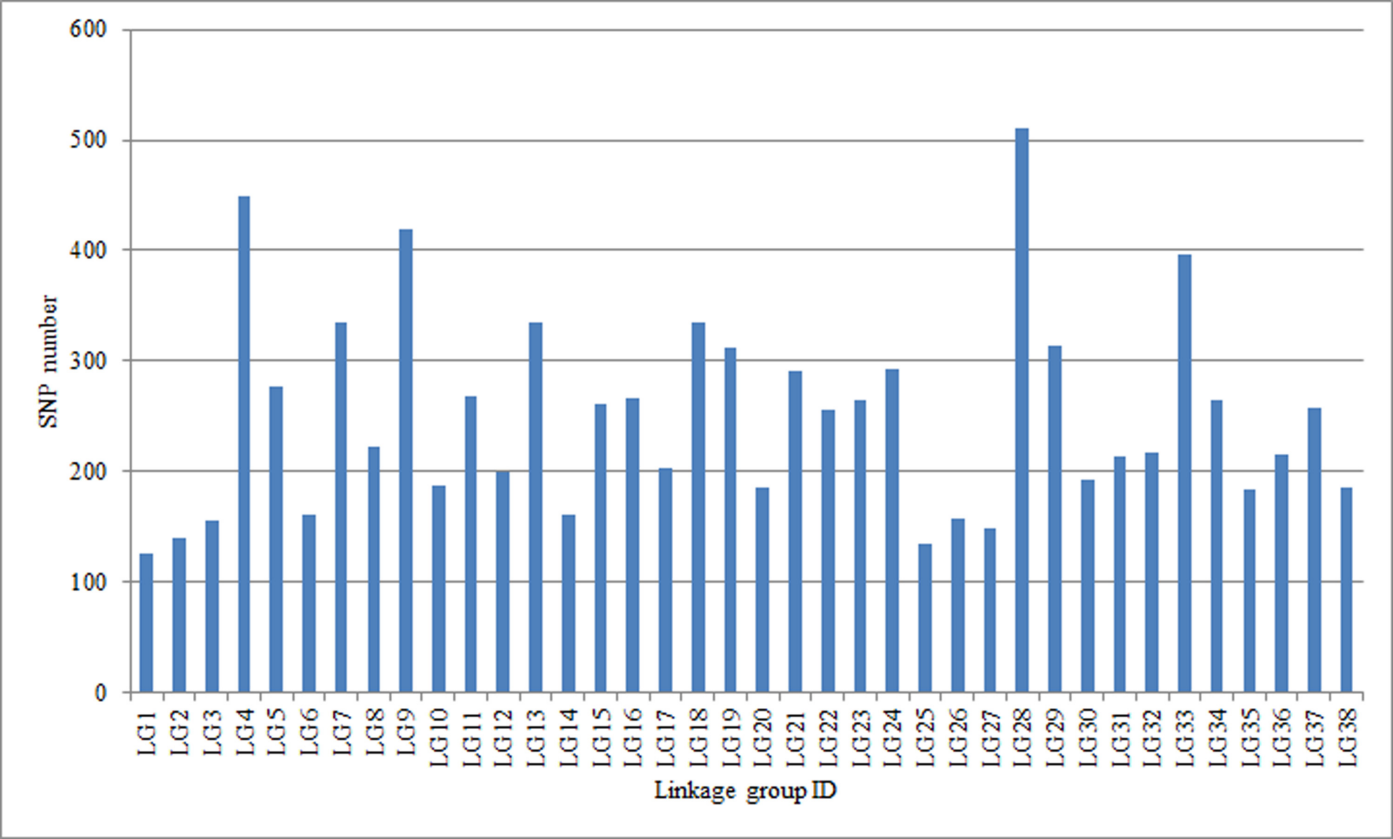
The number of single nucleotide polymorphism (SNP) markers on the linkage groups.

The MLOD values between the 6,744 SLAFs were calculated. Finally, 6,737 SLAFs were allocated into 38 LGs (S1 Fig). The linear arrangements of all SLAFs and genetic distances of adjacent SLAFs within each LG were analyzed using HighMap software. An integrated genetic map, 5,497.45 cM in total length and 0.82 cM in average length, was finally constructed (S1 Fig, Table 4). A total of 9,488 SNP markers were included on the map. The number of SNP markers in different LGs ranged from 125 to 511 on the map (Fig 5). The mean sequencing depth of the SLAFs on the map were 190.38-fold for the parents and 28.68-fold for the offspring. The basic characteristics of the LGs on the male map and the female map are listed in Tables 5 and 6, respectively.

**Table 4:** Basic characteristics of the linkage groups in the integrated genetic map of *S*. *matsudana*.

Linkage group ID	Marker number	Total distance (cM)	Average distance (cM)	Max gap
**LG1**	90	110.12	1.24	11.64
**LG2**	94	104.83	1.13	9.09
**LG3**	115	96.02	0.84	5.94
**LG4**	305	240.00	0.79	8.52
**LG5**	206	175.73	0.86	6.81
**LG6**	120	123.86	1.04	12.62
**LG7**	236	166.48	0.71	5.23
**LG8**	158	129.30	0.82	7.39
**LG9**	301	175.40	0.58	10.47
**LG10**	123	156.42	1.28	12.10
**LG11**	187	137.38	0.74	5.29
**LG12**	130	111.51	0.86	6.82
**LG13**	232	151.98	0.66	7.76
**LG14**	117	121.46	1.05	13.03
**LG15**	186	155.46	0.84	6.73
**LG16**	192	126.16	0.66	8.59
**LG17**	153	137.23	0.90	16.77
**LG18**	243	182.24	0.75	10.83
**LG19**	198	138.26	0.70	7.12
**LG20**	144	121.48	0.85	6.13
**LG21**	207	165.44	0.80	8.02
**LG22**	169	123.62	0.74	7.00
**LG23**	189	142.91	0.76	5.29
**LG24**	204	150.69	0.74	8.33
**LG25**	106	106.93	1.02	10.54
**LG26**	117	113.58	0.98	11.38
**LG27**	115	103.07	0.90	7.62
**LG28**	361	252.34	0.70	7.31
**LG29**	229	174.32	0.76	13.78
**LG30**	127	129.50	1.03	13.51
**LG31**	152	144.46	0.96	9.72
**LG32**	155	153.93	1.00	7.50
**LG33**	273	174.28	0.64	6.8
**LG34**	190	144.56	0.76	7.55
**LG35**	136	106.10	0.79	8.23
**LG36**	160	148.81	0.94	11.62
**LG37**	185	172.34	0.94	19.89
**LG38**	132	129.25	0.99	7.67
**Total**	6,737	5,497.45	0.82	19.89

**Table 5:** Basic characteristics of the linkage groups in the male genetic map of *S*. *matsudana*.

Linkage group ID	Marker number	Total distance (cM)	Average distance (cM)	Max gap
**LG1**	56	110.86	2.02	38.11
**LG2**	60	105.83	1.79	28.17
**LG3**	90	102.32	1.15	6.68
**LG4**	219	236.64	1.09	8.00
**LG5**	147	167.01	1.14	16.21
**LG6**	70	118.73	1.72	14.13
**LG7**	172	172.33	1.01	18.39
**LG8**	115	131.19	1.15	7.75
**LG9**	208	180.94	0.87	9.77
**LG10**	79	151.02	1.94	18.84
**LG11**	119	139.98	1.19	10.21
**LG12**	86	114.69	1.35	18.85
**LG13**	150	139.82	0.94	15.48
**LG14**	88	126.55	1.45	10.00
**LG15**	134	162.26	1.22	7.79
**LG16**	128	115.44	0.91	8.59
**LG17**	82	135.49	1.67	22.24
**LG18**	157	175.56	1.13	8.75
**LG19**	125	147.88	1.19	11.48
**LG20**	90	119.89	1.35	15.37
**LG21**	149	154.52	1.04	13.39
**LG22**	106	115.41	1.10	12.76
**LG23**	135	147.34	1.10	6.57
**LG24**	121	128.54	1.07	14.81
**LG25**	80	97.05	1.23	13.95
**LG26**	75	118.86	1.61	19.58
**LG27**	75	100.27	1.36	7.75
**LG28**	274	257.32	0.94	12.79
**LG29**	152	187.12	1.24	26.40
**LG30**	101	116.52	1.17	12.15
**LG31**	102	148.57	1.47	7.54
**LG32**	120	137.75	1.16	8.96
**LG33**	173	185.30	1.08	11.11
**LG34**	131	153.16	1.18	11.48
**LG35**	100	95.35	0.96	10.84
**LG36**	122	147.13	1.22	17.23
**LG37**	88	107.11	1.23	24.70
**LG38**	96	127.28	1.34	8.36
**Total**	4575	5,379.03	1.19	38.11

**Table 6:** Basic characteristics of the linkage groups in the female genetic map of *S*. *matsudana*.

Linkage group ID	Marker number	Total distance (cM)	Average distance (cM)	Max gap
**LG1**	40	88.33	2.26	17.65
**LG2**	51	101.24	2.02	30.95
**LG3**	34	79.09	2.40	13.41
**LG4**	116	235.45	2.05	27.65
**LG5**	79	176.43	2.26	19.64
**LG6**	56	113.76	2.07	35.06
**LG7**	85	159.07	1.89	15.55
**LG8**	56	126.89	2.31	15.48
**LG9**	126	164.63	1.32	17.63
**LG10**	52	160.00	3.14	30.11
**LG11**	87	130.64	1.52	12.79
**LG12**	58	97.02	1.70	9.92
**LG13**	117	149.81	1.29	7.76
**LG14**	41	110.40	2.76	18.17
**LG15**	74	148.66	2.04	14.03
**LG16**	85	115.78	1.38	16.93
**LG17**	85	135.91	1.62	20.66
**LG18**	109	187.89	1.74	23.04
**LG19**	92	123.69	1.36	16.21
**LG20**	67	121.00	1.83	14.82
**LG21**	73	174.22	2.42	24.69
**LG22**	79	116.95	1.50	12.13
**LG23**	76	129.90	1.73	18.39
**LG24**	114	157.13	1.39	40.36
**LG25**	35	110.54	3.25	23.00
**LG26**	54	108.29	2.04	16.76
**LG27**	53	105.87	2.04	20.62
**LG28**	134	240.13	1.81	24.53
**LG29**	102	158.89	1.57	21.35
**LG30**	34	128.89	3.91	29.00
**LG31**	71	140.36	2.01	38.66
**LG32**	49	168.08	3.50	24.51
**LG33**	146	161.68	1.11	10.21
**LG34**	70	132.87	1.93	14.81
**LG35**	46	116.34	2.59	17.65
**LG36**	52	123.91	2.43	17.63
**LG37**	111	171.97	1.56	28.12
**LG38**	48	119.30	2.54	17.27
**Total**	2857	5,291.01	1.88	40.36

### Evaluation of the Genetic Map

**Fig 6:**
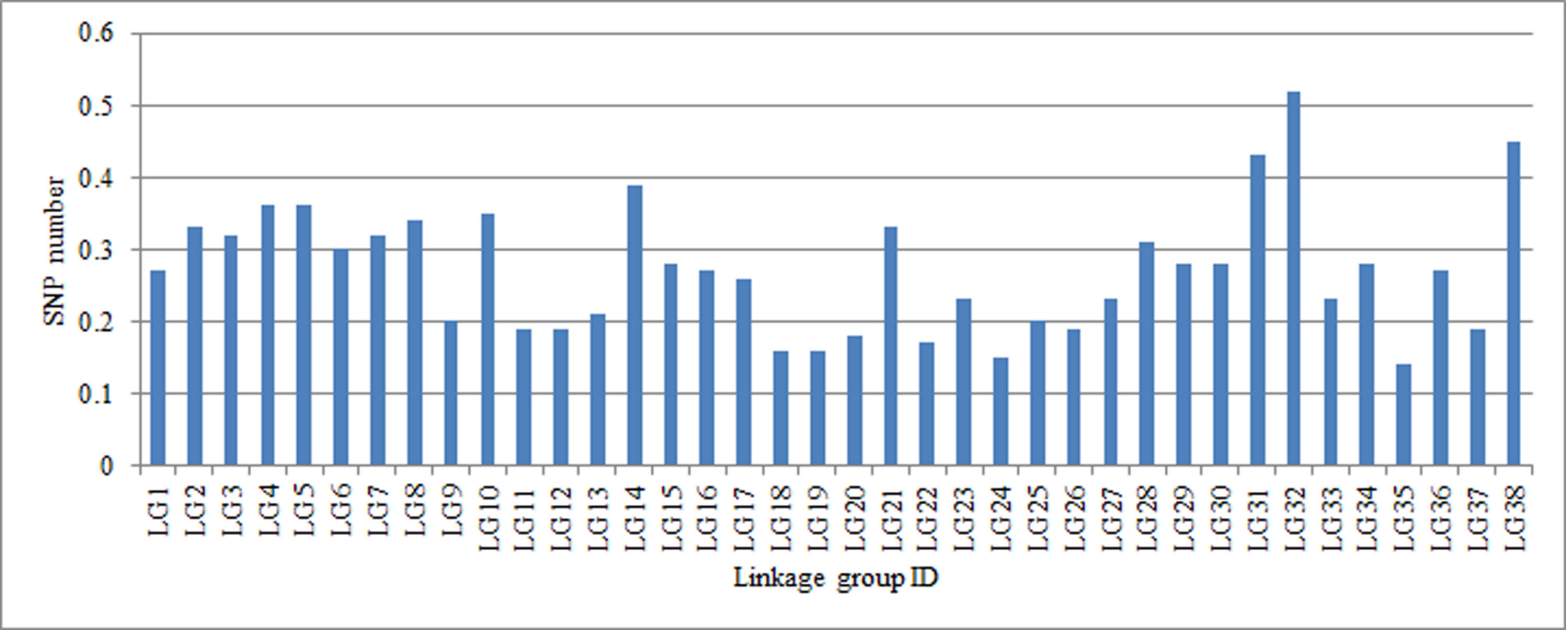
Missing percentage of markers in each of the linkage groups.

The genetic map of *S*. *matsudana* was evaluated using haplotype maps and heat maps. Haplotype maps were generated for each of the 195 F_1_ individuals using 6,737 SLAFs (S1 File). The recombination events of each individual on LGs of the integrated genetic map were displayed intuitively on the haplotype maps. As can be seen in S2 File, the majority of recombination blocks were clearly defined. The missing percentage of markers in each LG of the integrated genetic map ranged from 0.14% to 0.52% (Fig 6), which did not significantly affect the quality of the genetic map. It can also be seen that all LGs distribute uniformity. Heat maps were also generated to evaluate the quality of genetic map using pair-wise recombination values for the 6,737 mapped SLAFs (S2 File). Heat maps could reflect the recombination relationships between markers in each of the LGs and were used to find the potential ordering errors. It can be seen in S2 File that most of the LGs performed well in visualization, indicating that the markers were well-ordered in each LG. Consequently, the genetic map of *S*. *matsudana* was of high quality.

## Discussion

Willows show high-level variations in chromosome numbers, including diploid, tetraploid, hexaploid, and even dodecaploid [28, 29]. Variations in chromosome numbers of willows occur not only among species, but among varieties of the same species, which has led to difficulties in improving willows. *S*. *matsudana* is an important arbor willow species, certain varieties of which have become potential strategic resources in coastal developments of China because of their salt tolerances [9, 10]. The chromosome number of *S*. *matsudana* remains unknown, which has hindered studies on this species. To ensure accuracy in the subsequent experiments, ploidy levels of chromosome in the parents and the offspring were determined by comparing the peak values of their chromosomes with diploid *S*. *suchowensis* (2n = 2x = 38) [27, 30] using flow cytometry [26]. Our results showed that both the parents and the 15 randomly selected offspring were tetraploid (2n = 4x = 76) (Figs 1 and 2), which has provided a reference for genetic map construction of *S*. *matsudana*.

Genetic maps are the basis for QTL fine mapping of interesting traits, map-based gene cloning, and marker-assisted breeding. A suitable mapping population is the basis for successful construction of the genetic map. In this report, salt-sensitive “Yanjiang” and salt-tolerant “9901”, the intraspecific varieties of *S*. *matsudana*, were chosen as female and male parents, and their F_1_ hybrid population was used as the mapping population for genetic map construction. The segregation population may present with a large number of variations due to significant variations in the salt tolerance of the parents, which could facilitate QTL mapping of salt-tolerant related traits.

Genetic markers are powerful tools for genetic map construction. The selection of molecular markers is key for the success of genetic map construction. The molecular markers used to construct the genetic maps of willows included amplified fragment length polymorphism (AFLP), simple sequence repeat (SSR), and SNPs [13–15]. Among all the markers developed, SNPs are ideal markers for constructing the genetic map due to the advantages of abundance and even distribution across the genome. SNP molecular markers were used in this report, and are suitable for constructing the genetic map of *S*. *matsudana*.

The number of molecular markers is one of the indices for evaluating the quality of the genetic map. A small number of markers will lead to a map with low-density and large distance, thus decreasing the effective utilization of the map. Although previous genetic maps of willows have been utilized to a certain degree [13–15], the number of markers on the maps was only several hundred, which has hindered effective utilization. SNP markers are used widely in genetic map construction not only because of the advantages of the marker itself, but also because advances in high throughput sequencing technologies allow us to detect a large number of SNPs in a short time [17–20]. RAD-seq and SLAF-seq are among the sequencing technologies that can detect large-scale SNPs. RAD-seq is a reduced-genome sequencing technology that randomly digests genomic DNA with restriction enzymes followed by sequencing. SLAF-seq is a reduced-genome sequencing technology that digests genomic DNA with double restriction enzymes followed by sequencing of the paired-end reads with a specific length. Compared with RAD-seq, the largest advantage of SLAF-seq is its repeatability. SLAF-seq has been used to construct the genetic maps of a number of species in the last few years [21–24]. This report has also adopted the more advanced SLAF-seq technology, which was suitable for constructing the genetic map.

Using intraspecific varieties of tetraploid *S*. *matsudana*—“Yanjiang” and “9901” as parents, the randomly selected 200 F_1_ hybrid offspring as a mapping population, and the high-throughput SLAF-seq to construct the genetic map of *S*. *matsudana*, it can be seen from the map that a total of 277,333 SLAFs were obtained, of which 99,526 were polymorphic (Table 3). After filtering, the final number of SLAFs were 6,737 for the integrated genetic map (Table 4), 4,575 for the male genetic map (Table 5), and 2,857 for the female genetic map (Table 6), respectively. The integrated genetic map consisted of 38 linkage groups with total and average genetic distances of 5,497.45 cM and 0.82 cM, respectively (S1 Fig, Table 4). The number of SLAFs in each LG of the integrated genetic map ranged from 90 to 361. A total of 9,488 SNP markers were included on the integrated map. The map was of high quality based on evaluation of typlotype sources and linkage relationships (S1 and S2 Files). These results showed that SLAF-seq is a reliable technology to detect large-scale SNPs and construct genetic maps.

The intraspecific F_1_ offspring used for map construction were derived from two tetraploid parents. Previous cytological and marker-segregation analyses show that tetraploid willows are allotetraploid, a group of tetraploids with two sets of chromosomes, each from a different ancestral species [15]. Because allotetraploids usually undergo disomic inheritance, approaches for their linkage analysis can be directly borrowed from those for diploids. However, these approaches do not characterize the difference of meiotic pairing occurring between homologous chromosomes from that between homeologous chromosomes, and therefore, the genome structure, organization and evolution of allotetraploids. By implementing the preferential pairing factor, Wu and colleagues have developed a series of statistical models that can identify the difference between homologous pairing and homeologous pairing [31, 32]. These models can be used to analyze our tetraploid marker data and identify the degree of the preferential pair of homologous chromosomes over homeologous chromosomes. This information helps to gain new insight into the evolution of *Salix* genomes.

In nature, the meiotic recombination may differ between the two sexes, a phenomenon called heterochiasmy [33]. Wu et al. [34] modified a linkage analysis model to estimate and test heterochiasmy using marker data generated from a controlled cross. The application of this model provides additional insight into the genome-wide occurrence of heterochiasmy in *Salix*. The high-density genetic map constructed in this study provides a basis for QTL mapping, map-based gene cloning, and molecular breeding of willows, and gives important information for selection, breeding, protection, and utilization of salt-tolerant willow varieties; furthermore, the map will advance the genetic improvement of willows. Since no whole genomes of willows are available, the 38 linkage groups could not be further divided into two sets. This genetic map will be improved once the whole genome of willows is available.

## Conclusion

Using the intraspecific F_1_ hybrid population of salt-tolerant and salt-sensitive varieties of tetraploid *S*. *matsudana* as the mapping population, a total of 277,333 SLAFs were obtained, among which 99,526 were polymorphic, and 42,321 of the polymorphic markers could be used as potential markers for constructing the genetic map. After filtering the low quality markers and unsuitable markers for the F_1_ population, 6,737 high quality SLAFs were used to construct a high quality genetic map. The genetic map consisted of 38 LGs, with total and mean genetic distances of 5,497.45 cM and 0.82 cM, respectively. The mean sequencing depth of SLAFs for the parents and offspring were 190.38-fold and 28.68-fold, respectively. The results from this study will provide a basis for fine mapping of salt-tolerant related QTLs and molecular breeding of *S*. *matsudana*.

## Supporting Information

S1 Fig High-density genetic map of *S*. *matsudana*. (PDF)

S1 File Haplotype maps of the genetic map. Green represents “9901”, blue represents “Yanjiang”, white means the parent could not be estimated, grey represents deletions.
(RAR)

S2 File Heat maps of the genetic map. Each cell represents the recombination rate of two markers. Yellow indicates a lower recombination rate and purple a higher one.
(RAR)
