# Supplementary figures and images for: A High-Density Genetic Map of Tetraploid Salix matsudana Using Specific Length Amplified Fragment Sequencing (SLAF-seq)
Source: PLoS One. 2016 Jun 21;11(6):e0157777. doi: 10.1371/journal.pone.0157777 (PMC4915623; doi:10.1371/journal.pone.0157777)

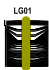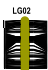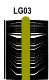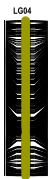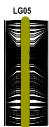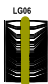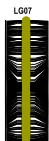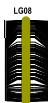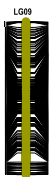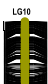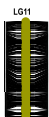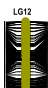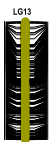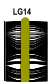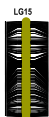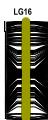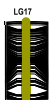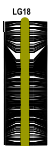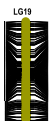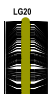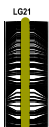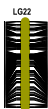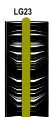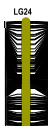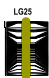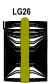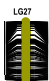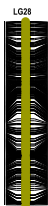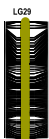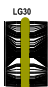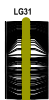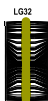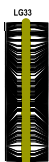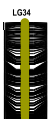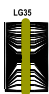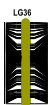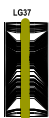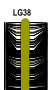

Supplement: S1 Fig — (PDF) [file pone.0157777.s001.pdf]
